# Two year follow up of supercapsular percutaneously assisted total hip arthroplasty

**DOI:** 10.1186/s12891-021-04351-0

**Published:** 2021-05-24

**Authors:** Andrew Kay, Derek Klavas, Varan Haghshenas, Mimi Phan, Daniel Le

**Affiliations:** 1 Department of Orthopedics and Sports Medicine, Houston Methodist Hospital, 6445 Fannin St. Suite 2500, Houston, TX 77030 USA; 2 Texas A&M College of Medicine, 8447 Bryan Rd, Bryan, TX 77807 USA; 3 Department of Orthopedics and Sports Medicine, Houston Methodist Willowbrook Hospital, 18220 TX-249, Houston, TX 77070 USA

**Keywords:** Superpath, Supercapsular percutaneously assisted total hip arthroplasty, Total hip arthroplasty, Dislocation rate, Prosthetic hip dislocation

## Abstract

**Background:**

Dislocation after primary total hip arthroplasty (THA) has an incidence of 2–3%. Approximately 77% of dislocations occur within the first year after surgery. The SuperPATH technique is a minimally invasive approach for THA that preserves soft tissue attachments. The purpose of this study is to describe the dislocation rate at 1 year after SuperPATH primary THA.

**Methods:**

All elective primary THAs performed by the senior author using the SuperPATH approach. Exclusion criteria were acute femoral neck fracture, revision surgery, or malignancy. There were 214 of 279 eligible patients available for telephone interviews (76.7%). Medical records were reviewed for secondary outcomes including early and late complications, cup positioning, distance ambulated on postoperative day one, discharge destination, and blood transfusions.

**Results:**

Mean age at surgery was 64 ± 10.8 years and mean time to telephone follow up was 773 ± 269.7 days. There were 104 female and 110 male patients. There were zero dislocations reported. Blood transfusions were performed in 3.7% of patients, and 75.7% were discharged to home at an average of 2.3 ± 1.0 days. Cup position averaged 43.6 ± 5.2° abduction and 20.9 ± 6.2° anteversion, with an average leg length discrepancy of 3.6 ± 3.32 mm. Complications included three intraoperative calcar fractures, one periprosthetic femur fracture, one early femoral revision, three superficial infections, and one instance of wound necrosis.

**Conclusion:**

SuperPATH approach is safe for use in primary THA resulting in a low dislocation rate.

## Background

Minimally invasive surgical (MIS) approaches for elective THA have become increasingly popular due to the potential for decreased muscular damage, pain, blood loss, and time to mobilization [1–4]. The supercapsular percutaneously-assisted total hip (SuperPATH®, MicroPort Orthopedics Inc., Arlington, TN, USA) is a MIS approach that shares some similarities to the traditional posterior approach [5, 6]. The hip is approached through the interval between the Gluteus Medius and Piriformis, as well as through a distal percutaneous portal. The short external rotator muscles and Iliotibial band are not violated, the hip is not dislocated, thus theoretically reducing the risk of postoperative dislocation [7–9]. The small incision and overall tissue-sparing nature of this approach has been previously reported to allow for decreased time to ambulation, length of stay, 30-day readmission rates, in-hospital costs, and blood loss [10–12].

Dislocation is a serious complication of total hip arthroplasty (THA), with a reported incidence between 2 and 3% after primary THA, and it remains one of the most common reasons for revision surgery [1–11]. Approximately 77% of dislocations occur within the first year, and up to 50% in the first 3 months [13–15]. The incidence of dislocation is affected by various factors including cup position, head size, soft tissue tension, spinopelvic disease and possibly surgical approach [16–32]. While older studies demonstrated dislocation rates as high as 9–13% with the posterior approach, recent studies including a meta-analysis of 13,000 patients have demonstrated dislocation rates closer to that of other approaches [33–38]. Theoretically, because of the reduced muscular, tendinous, and capsular dissection, it may be possible that the SuperPATH THA is less prone to dislocation. However, due to limited visualization secondary to the small incision, it is possible that that surgeons may not achieve optimal component position, thus potentially predisposing the hip to dislocation.

The purpose of the current study was to evaluate early of patients undergoing primary, elective THA using the SuperPATH approach. Outcomes included days to ambulation, distance ambulated on postoperative day one (POD1), surgical time from incision to dressing, estimated blood loss (EBL), hemoglobin decrease on POD1, perioperative blood transfusions, discharge disposition, radiographic outcomes, and complications. Additionally, complications including repeat surgery and dislocation were assessed.

## Methods

IRB approval was obtained for a retrospective chart review and telephone interviews. All consecutive elective, primary SuperPATH THAs performed by the senior author at least 1 year prior to the study date were reviewed retrospectively, beginning with the surgeon’s first case in practice on 11/21/2013 and ending on 12/5/2016. Exclusion criteria were a diagnosis of acute femoral neck fracture, revision surgery, other THA approach, metastatic disease, or surgery performed within 1 year of the study. The senior author was trained in the SuperPATH approach during fellowship, and uses it exclusively for elective primary THA, as well as for THA for femoral neck fractures. However, in contrast to the originally described technique, the treating surgeon in this study completely releases the piriformis tendon intraoperatively to gain better exposure [6]. The surgeon additionally uses a mechanical guide for acetabular alignment that is based off-of a preoperative CT scan (HipXpert®, Surgical Planning Associates, Inc., Boston, MA, USA). Postoperatively, there are no specific hip precautions. Patients worked with physical therapy (PT) starting on either the day of surgery or POD1.

Electronic medical records were reviewed to collect patient demographics and results. Medical records and post-operative radiographs were reviewed to assess for dislocation and secondary outcomes. Standing radiographs taken in clinic 3 weeks post-operatively were reviewed and analyzed with commercially available templating software (Traumacad®, Brainlab, Munich, DE) to measure cup abduction, anteversion, and leg length discrepancy, a method that has been found to correlate well with CT-based measurements of implant position [39–41]. Number of dislocations were confirmed through telephone interviews with the patients 1 year or more after surgery. During telephone interviews, patients were also asked if the operative hip had experienced any dislocations, secondary surgery or any other complications in order to assess for any treatments that may have been rendered at outside institutions.

Descriptive statistics were calculated for patient demographics, perioperative data, and follow-up data. Continuous variables were presented as mean (range) and categorical variables presented as whole integers with incidences. Component measurements were described as mean (standard deviation). Data was analyzed with Microsoft Excel software (Microsoft Corporation, Redmond, WA, USA).

## Results

### Patient population

There were 279 primary THAs performed on 254 patients during the study period, of which 214 cases met inclusion criteria and were available for telephone follow-up (76.7%). The mean patient age was 64 ± 10.8 years (range 26–93). The mean time to telephone follow-up was 773 ± 269.7 days (range 368–1449). There were 104 female patients and 110 male patients. Ninety-two operations were left-sided and 122 right. Mean BMI was 29.5 ± 5.9 (range 17.3–55.7). Preoperative diagnosis was primary osteoarthritis in 172 patients, avascular necrosis in 18 patients, acetabular or proximal femoral dysplasia with degenerative changes in 14 patients, posttraumatic arthritis in 7 patients, rheumatoid arthritis in 2 patients, and femoral neck fracture nonunion in one patient. Demographics of study population are seen in Table 1.

**Table 1:** Demographics of the study population

Demographics	
**Mean Age, yrs (range)**	**64 ± 10.8 (26–93)**
Female	110 (51.4%)
Male	104 (48.6%)
**Laterality, n (%)**	
Right	122 (57.0%)
Left	92 (43.0%)
**Mean BMI, kg/m^2 (range)**	29.5 ± 5.9 (17.3–55.7)
**Diagnoses, n (%)**	
Primary OA	172 (80.4%)
AVN	18 (8.4%)
Dysplasia	14 (6.5%)
Post-traumatic arthritis	7 (3.3%)
Rheumatoid arthritis	2 (0.9%)
Femoral neck nonunion	1 (0.5%)

*Abbreviations*: *BMI* Body mass index, *OA* Osteoarthritis, *AVN* Avascular necrosis

### Perioperative measures

All but three patients were ambulatory on POD1, of whom one had experienced progressively declining ambulatory function prior to surgery secondary to a traumatic brain injury. Average distance ambulated on POD1 was 181 ± 152.5 ft (range 0–800) reported through physical therapy notes. Surgical time averaged 136 ± 40.5 min (range 77–475). The first 20 cases averaged 176 min, and the last 20 averaged 111 min. EBL averaged 321 ± 230 cc (range 50–1700). Hemoglobin dropped by an average 1.6 ± 1.0 g/dL during surgery (range 0.2–5.9). Intraoperative Cell Saver blood salvage was initially used, but abandoned midway through the study period due to lack of perceived benefit by the treating surgeon. Spinal anesthesia and tranexamic acid were also used to help reduce blood loss. A total of eight patients (3.7%) needed blood transfusions in the perioperative period. One hundred sixty-two patients (75.7%) were discharged to home, four to home with home health, 44 to inpatient rehabilitation, and four to skilled nursing facilities. Time to discharge averaged 2.3 ± 1.0 days (range 1–8), with 72.4% discharged on POD1. Perioperative outcomes are seen in Table 2.

**Table 2:** Perioperative outcomes

Perioperative outcomes	
**Mean ΔHgb, g/dL (range)**	1.6 ± 1.0 (0.2–5.9)
**Mean Surgical time, min (range)**	136 ± 40.5 (77–475)
**Mean EBL, mL (range)**	321 ± 230 (50–1700)
**Transfusion, n (%)**	8 (3.7)
**Mean Ambulation POD1, ft (range)**	181 ± 152.5 (0–800)
**Mean Hospital LOS, days (range)**	2.3 ± 1.0 (1–8)
**Discharge destination, n (%)**	
Home	162 (75.7)
Home w/ home health	4 (1.9)
IPR	44 (20.6)
SNF	4 (1.9)

*Abbreviations*: *Hgb* Hemoglobin, *EBL* Estimated blood loss, *POD* Post-operative day, *LOS* Length of stay, *IPR* Inpatient Rehab, *SNF* Skilled nursing facility

### Components and positioning

**Fig. 1:**
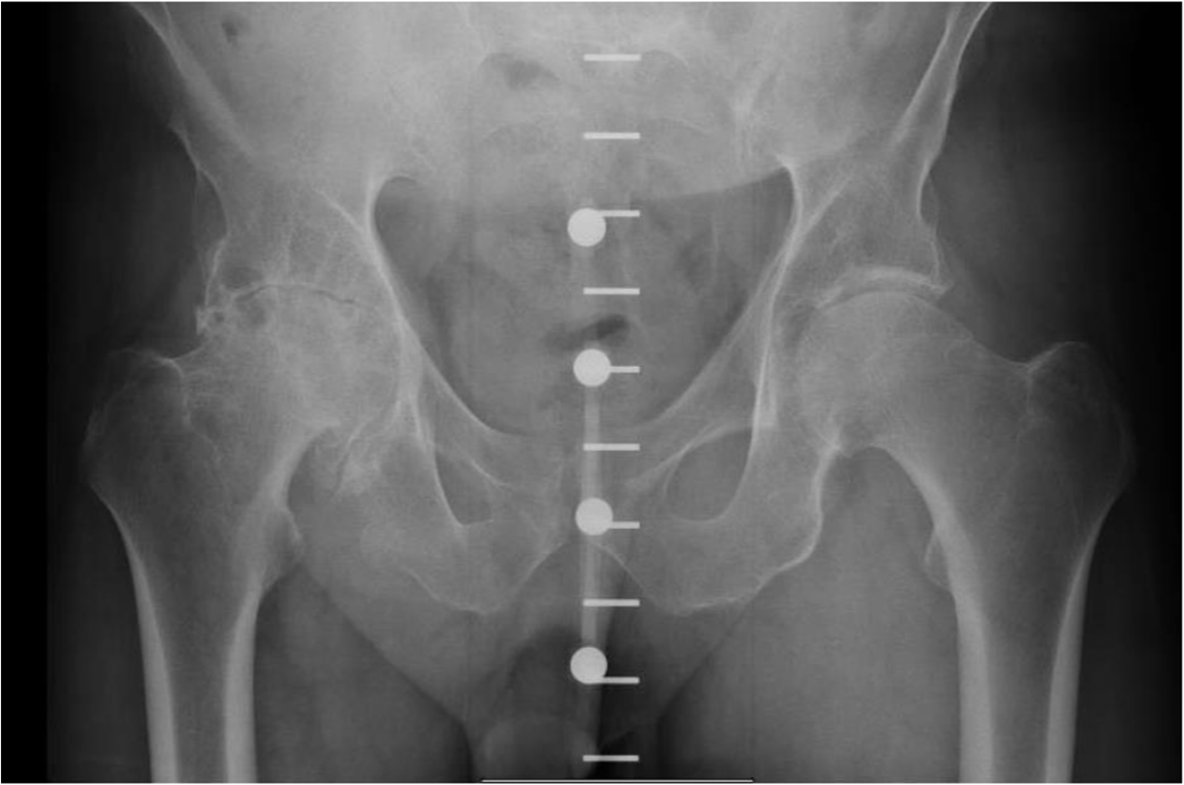
Pre-operative radiograph from one of our study participants

**Fig. 2:**
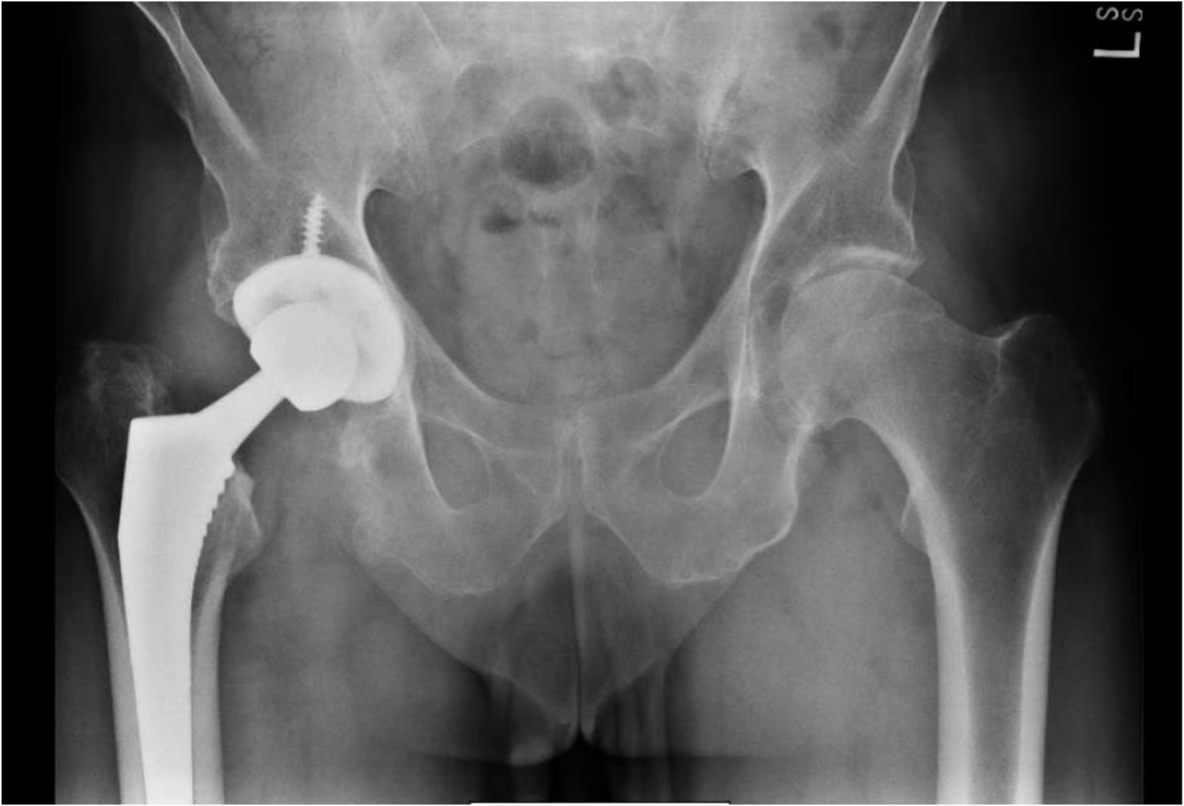
Post-operative radiograph from one of our study participants. For a live surgical demonstration please see the following link: https://www.vumedi.com/video/superpath-the-direct-superior-portal-assisted-total-hip-approach-live-surgery/

Most implants were non-modular Microport stems, cups, and polyethylene liner. One Link revision stem was used after an intramedullary nail removal. One patient received a modular DePuy S-ROM® implant due to proximal femoral dysplasia. The majority of components were press-fit except for two patients who received cemented femoral component due to intraoperative calcar fractures. Cup position averaged 43.6 ± 5.2° of abduction (range 29.0–66.0) and 20.9 ± 6.2° of anteversion (range 5.0–53.0). Leg length discrepancy averaged 3.6 ± 3.3 mm (range 0.0–16.0). As far as head size, four patients (1.9%) received a 28 mm head, two (0.9%) received 30 mm heads and the remainder (97.2%) received 36 mm or higher. Preoperative and postoperative radiographs of one of the study participants are seen in Figs. 1 and 2 respectively.

### Complications

There were zero dislocations throughout the study period. Intraoperatively, there were three calcar fractures (1.4%), and one patient needed an immediate return to the operating room (OR) from post-anesthesia care unit (PACU) after a postoperative radiograph revealed a loose piece of bone interposed in between the prosthetic head and polyethylene liner. There were two operations to revise components. One patient sustained a periprosthetic femur fracture 21 days after the index surgery and underwent revision of the femoral component. Another underwent femoral component revision 19 months after the index procedure for aseptic loosening. There was one irrigation and debridement of a superficial abscess, three suture reactions treated conservatively, and one wound revision performed in the office for skin necrosis. Femoral stem subsidence of 2 mm was noted on one patient at the initial postoperative visit, but thereafter remained stable and no further treatment was necessary. There were no deep infections.

## Discussion

This series demonstrates the successful use of the SuperPath approach for THA, with most patients ambulatory and POD. Additionally, there were zero dislocations and zero deep infections in 214 patients 1 year after surgery.

Rapid time to ambulation is a theoretical advantage of MIS THA approaches. The vast majority of patients in the present series were ambulatory on POD1, and most (75.7%) were discharged home without home health. Multiple studies have demonstrated similar recovery at home as compared to rehabilitation or skilled nursing facilities after TJA, with or without another person living in the home [42–48]. Bozic et al. reported that post discharge payments account for 36% of total Medicare payments for total joint arthroplasty, of which 70% is consumed by the 49% of patients who are discharged to post-acute care facilities [49]. By allowing the majority of patients to discharge home, the SuperPATH approach may enable significant cost savings, findings that were supported by a recent economic analysis by Chow and Finch [11].

EBL was 321 cc. Although intraoperative surgeon EBL is often underestimated [50], patients overall experienced small decreases in hemoglobin after surgery (1.6 g/dL), as well as a low rate of intraoperative and postoperative blood transfusions (3.7%). A recent analysis of the Nationwide Inpatient Sample of 2,087,423 THAs found that the rate of allogenic blood transfusion increased from 11.8% in 2000 to 19.0% in 2009 [51]. A prospective study of 92 patients randomized to either SuperPATH or the posterior approach THA demonstrated a decreased rate of transfusions with SuperPATH, although the results were not statistically significant [52]. Allogenic blood transfusions have been associated with increased risk of infection after total joint arthroplasty, and the SuperPATH approach may help reduce this risk by reducing blood loss [53].

This series is from the beginning of the senior author’s career. Overall operative time averaged 136 min from skin incision to dressing application, but decreased 65 min from the first 20 cases to the last 20, which may represent the effect of the initial learning curve. Rasuli and Gofton found that operative time continued to significantly decrease with the SuperPATH approach even at the 50th case, implying a longer learning curve that may require extensive experience to become proficient [54]. A recent retrospective analysis of the National Surgical Quality Improvement Program database reported an average operative time of 94 min in 103,000 THAs, which is shorter than reported in the present series [55]. However, operative times did not translate into an unacceptably high rate of infection nor complications in this series.

Adequate visualization is an inherent challenge in MIS THA. There is some evidence that the risk of intraoperative periprosthetic fracture is elevated with MIS approaches for elective THA [56, 57]. However, the present study demonstrated an incidence of 1.4%, which is lower than the 2.95–10.6% reported in other large series [58–61]. The three intraoperative fractures in the present series were all treated successfully using cerclage cables inserted through a smaller secondary incision without compromising the short external rotators. The only other complication attributable to decreased visualization was a return to the OR from PACU after a postoperative radiograph demonstrated a previously unrecognized piece of bone interposed between the prosthetic head and cup.

Prosthetic hip dislocation is one of the most common early complications after primary THA, and may be influenced by factors including surgical approach and cup position [20, 31, 32, 62–73]. The present study shows that the SuperPath approach may present an opportunity for the surgeon to further reduce dislocation incidence below the reported rates of 2–3% [13, 16–19, 33, 74–78]. Additionally, the present study demonstrates the ability of CT-assisted navigation to achieve adequate cup position within the classic safe zone as described by Lewinnek et al., despite the decreased visualization that accompanies a smaller incision [31]. It should be noted that dislocation rates have decreased in recent years due to multiple factors such as the increased popularity of larger femoral heads, capsular repair, increased offset stems, and the impact of surgical approach remains controversial [79–85].

The present study is limited by several factors. Most notably, the retrospective data comes from a single surgeon who routinely uses the SuperPATH approach at a single institution. Without a control group undergoing a different approach, it is difficult to isolate the effect of the approach itself. Additionally, the number of total patients was relatively small, and prior analysis has shown that a sample size of 3720 patients would be needed to detect a 2% difference in dislocation rates of two different methods of THA with 80% power, leaving our study underpowered [86]. Thus, outcomes may not be generalizable to the broader patient population. Furthermore, telephone and email follow up was 76.7%, and although the medical records were examined for dislocations or other complications, it is possible that some patients unavailable for the survey experienced a dislocation that was treated at an outside facility. However, we note that of the patients unavailable for the survey, review of the medical records found none that had undergone treatment for a prosthetic joint dislocation at our hospital system.

## Conclusions

The present study demonstrates good early results for THA performed through the SuperPATH approach by a single surgeon in the early stages of his career. Despite the limited visualization that accompanies a small incision, overall complication rates were low, good cup position was achieved, and there were no dislocations nor deep infections. We do note that the senior author was trained in the SuperPATH approach during fellowship and these results may not be representative of the early experience of surgeons who are already facile with alternative approaches. Future, larger prospective research to compare outcomes to other approaches is needed.

## Data Availability

The datasets used and/or analyzed during the current study available from the corresponding author on reasonable request.

## Abbreviations

THA: Total Hip Arthroplasty
SuperPATH: Percutaneously Assisted Total Hip Arthroplasty
PACU: Post Anesthesia Care Unit
OR: Operating Room
MIS: Minimally Invasive Surgery
TJA: Total Joint Arthroplasty
EBL: Estimated Blood Loss
CT: Computed Tomography
PT: Physical Therapy
